# Endoscopic Endonasal Surgery for the Resection of a Cavernous Hemangioma with a Sellar Extension

**DOI:** 10.7759/cureus.3663

**Published:** 2018-11-30

**Authors:** Nicolas K Khattar, Shawn WC Adams, Alexandra S Schaber, Andrew C White, Mohammed Al Ghamdi, Rob T Hruska, Jesse J Savage, Richard K Downs, Eyas M Hattab, Brian J Williams

**Affiliations:** 1 Neurological Surgery, University of Louisville School of Medicine, Louisville, USA; 2 Radiology, University of Louisville School of Medicine, Louisville, USA; 3 Pathology, University of Louisville School of Medicine, Louisville, USA; 4 Neurological Surgery, Goodman Campbell Brain and Spine / Indiana University, Bloomington, USA

**Keywords:** cavernous hemangioma, sellar lesion, endoscopic endonasal surgery

## Abstract

Cavernous hemangiomas with an intrasellar extension are very rare, generally benign lesions that manifest by the compression of nearby structures. The presenting symptoms usually range from visual disturbances to an endocrine imbalance. Occasional extension into the cavernous sinus has been reported, which can cause cranial nerve compression. We present the case of a 69-year-old man presenting with facial pain and decreased libido. On investigation, a lesion was identified and the parasellar region was homogeneously hyper-intense on gadolinium-enhanced magnetic resonance imaging (MRI). Endoscopic endonasal surgery remains one of the favored approaches for the resection of sellar lesions. Such pathology needs to remain on the neurosurgeon’s differential diagnosis, making an intraoperative frozen section of these lesions a useful tool in the surgeon's armamentarium, to guide further surgical resection.

## Introduction

Sellar masses represent around 14%-18% of all brain tumors [1]. Vascular malformations involving the sella are exceedingly rare and have seldom been reported in the literature [2-4]. Such lesions should be considered in the differential diagnosis of the pituitary prior to resection [5]. We present the case of a patient with a contrast-enhancing cavernous hemangioma with sellar extension with an appearance consistent with a recurrent Rathke’s cleft cyst.

## Case presentation

Clinical history

**Figure 1:**
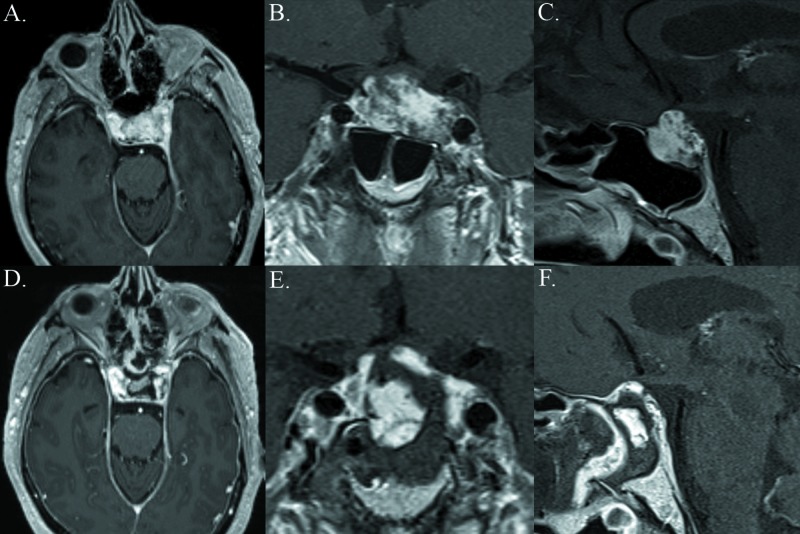
Gross total resection of the sellar cavernous hemangioma Upper: Preoperative axial, coronal, and sagittal T1-weighted MRI studies obtained after the administration of Gd, demonstrating a sellar contrast-enhancing recurrent mass measuring 2.7 cm x 1.9 cm x 1.6 cm extending into the left cavernous sinus Lower: Postoperative axial, coronal, and sagittal T1-weighted MRI studies obtained after gross total resection with EES. Fat graft was used under the vascularized nasoseptal flap. MRI: magnetic resonance imaging; EES: endoscopic endonasal surgery

The patient is a 69-year-old man who presented with new-onset facial pain. He reported a decrease in libido and a history of nocturia. The patient had a history of prior trans-sphenoidal endoscopic endonasal pituitary surgery for a possible Rathke’s cleft cyst six months prior to presentation. Gadolinium-enhanced magnetic resonance imaging (MRI) of the brain revealed a 2.7 x 1.9 x 1.7 cm recurrence of the previously resected sellar mass (Figure 1). The preoperative pituitary panel did not show any abnormalities. The patient was offered endoscopic endonasal surgery for the resection of the recurrent mass.

Surgical intervention

The patient underwent a resection of the lesion using purely endoscopic endonasal surgery (EES) with the two-surgeon technique [6]. Revision exposure was performed to expand the opening into the pituitary region. A dural opening was performed and several biopsy specimens were sent for intra-operative frozen section, which showed a pathological diagnosis of normal vasculature. The vascular mass was then resected until the exposure of the normal pituitary gland tissue. Adequate circumferential decompression was ensured. The routine skull base reconstruction consisted of multiple inlays of collagen matrix covering the entire bony defect. A fat graft was also applied as supplemental biological packing underlying the vascularized flap, which covered the entire construct.

Post-operative course

The patient sustained an intraoperative cerebrospinal fluid (CSF) leak and a lumbar drain remained in place for four days postoperatively with no evidence of leakage. The patient did not sustain any endocrinopathies or cranial neuropathies, remained stable, and was discharged five days postoperatively. The patient was followed up in clinic around one month following surgery and did not experience any additional deficits.

## Discussion

Cavernous hemangiomas with an intrasellar extension are extremely rare lesions of the sella turcica with very few reported cases in the literature [2-4]. These cases represent an obvious diagnostic challenge to any neurosurgeon. Presenting symptoms are usually due to a local mass effect on adjacent structures and limit the diagnosis to an expansive compressive lesion of the sella. In some patients, the presenting symptom was “chiasmal apoplexy,” which is associated with an abrupt headache, diminished visual fields, and an acute decrease in visual acuity [2]. In other patients, pituitary endocrine dysfunction is the presenting symptom [4]. Our patient presented with an amalgam of pituitary dysfunction and compression of the cavernous sinus and cranial nerves. Intracranial hemangiomas occur in 0.5% to 1% of the population and represent 5%-10% of all intracranial vascular malformations. These lesions often occur in the cerebral hemispheres and typically present with seizures and hemorrhages [7].

Magnetic resonance imaging typically shows hypo- or iso-intensity on T1-weighted imaging and significant hyper-intensity on T2 imaging with homogenous enhancement following the administration of gadolinium [3,8]. The most common type of cavernous hemangioma would not be evident on vascular studies, given that most hemangiomas are relatively low-flow lesions. Differentiating between pituitary adenomas and hemangiomas can be a challenging undertaking, even for experienced neuroradiologists [3].

Surgical resection is the treatment of choice for symptomatic hemangiomas to relieve the mass effect on adjacent structures [3,9]. During the operative intervention, if the consistency or vascularity of the lesion diverges from expected results, an intraoperative frozen section with neuropathological consultation will help guide the remainder of the surgical plan [3].

**Figure 2:**
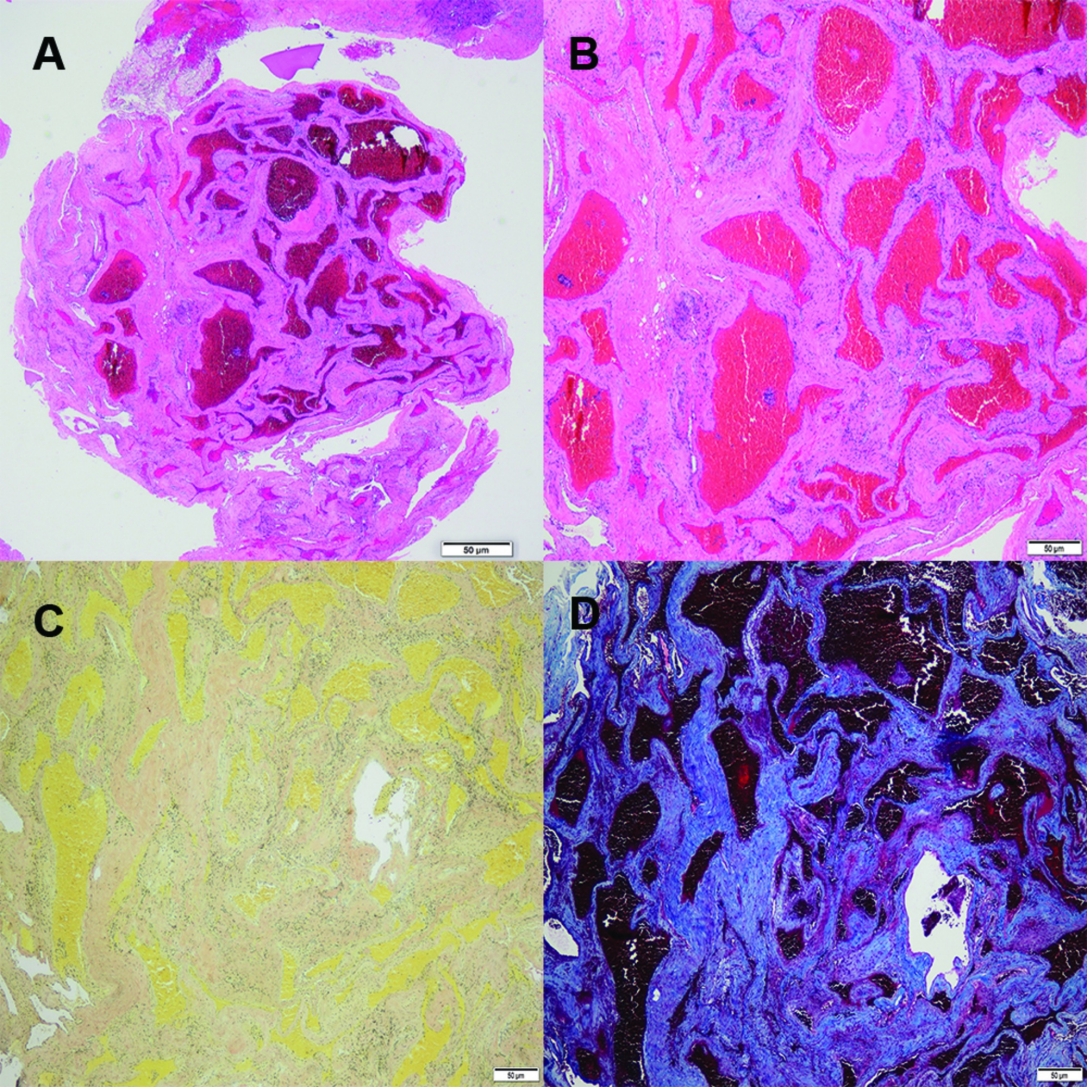
Sellar cavernous hemangioma pathological characteristics A. Low power magnification H&E stain of the hemangioma showing large, cystically dilated vessels with thin walls; B. High power magnification H&E stain; C. Elastin staining with no significant presence of elastin in the vessel walls of the arteries and arterioles of the hemangioma; D. Trichrome stain showing significant associated fibrosis with the hemangioma H&E: haemotoxylin and eosin

Histopathologically, cavernous hemangiomas appear as dilated vascular spaces, sclerotic vessels without intervening neural tissue, and intravascular thrombi suggesting slower blood flow [10]. Endoscopic endonasal surgery remains an optimal approach to the sella region and should be considered in the resection of any lesion [6] (Figure 2).

## Conclusions

Cavernous hemangiomas with a sellar extension are exceedingly rare lesions that become symptomatic due to the compression of nearby structures. Surgical resection is the treatment of choice for symptom relief and complete cure. This entity should be considered in the surgeon's differential diagnosis during management and work-up when considering an approach to this pathology.
